# Heinrich Wieleitner (1874–1931) und *Die Geburt der modernen Mathematik* – Wissenschaftskommunikation und Mathematikgeschichtsschreibung in der Weimarer Zeit

**DOI:** 10.1007/s00048-022-00356-5

**Published:** 2023-02-15

**Authors:** Maria M. Remenyi

**Affiliations:** Interdisziplinäres Zentrum für Wissenschafts- und Technikforschung, Bergische Universität Wuppertal, Gaußstr. 20, 42119 Wuppertal, Deutschland

**Keywords:** Historiografie der Mathematik, Wissenschaftskommunikation, Weimarer Zeit, Heinrich Wieleitner, Mathematik und Medien, Historiography of mathematics, Science communication, Scholarly communication, Weimar culture, Heinrich Wieleitner, Mathematics and the media

## Abstract

Am Beispiel des Mathematikhistorikers Heinrich Wieleitner untersucht der Aufsatz zum einen die enge personelle und inhaltliche Verflechtung von Mathematikgeschichtsschreibung, Mathematikdidaktik und Mathematikkommunikation und verweist so auf die vielfältigen Wechselbeziehungen zwischen externer und interner Wissenschaftskommunikation.

Die Fallstudie eröffnet darüber hinaus unter Einbeziehung medienhistorischer Aspekte neue Einblicke in die personelle, institutionelle und inhaltliche Struktur mathematischer Öffentlichkeitsarbeit im 20. Jahrhundert. Der Fokus liegt dabei auf der Weimarer Zeit, deren kulturelle Debatten das Selbstverständnis der Mathematik nicht nur auf besondere Weise herausforderten. Sie erwiesen sich, wie im Folgenden gezeigt wird, unter den Bedingungen einer sich verändernden Medienlandschaft auch als Chance für eine publizistisch erfolgreiche Selbstdarstellung der Disziplin.

## Abstract (en)

By taking the work and life of the historian of mathematics Heinrich Wieleitner as an example, this study aims to highlight the many interrelations between the historiography of mathematics, mathematics education, and science communication in mathematics.

By integrating aspects of the history of media, this case study also explores mathematical public relations work in the 20th century and draws attention to the important persons, institutions and contents. The focus is on the Weimar period, in which the self-understanding of mathematics was challenged in different ways by far-reaching cultural debates. The article demonstrates that as a consequence of a changing media landscape, Weimar culture turned out to be a suitable environment for the successful self-presentation of mathematics.

## Einführung

In der umfangreichen Forschungsliteratur zu den Wechselbeziehungen zwischen Wissenschaft(en) und Öffentlichkeit(en) kommt der Mathematik als eigenständiger Disziplin meist nur eine untergeordnete Rolle zu (Bauer & Howard 2013: 9). Auf den ersten Blick nachvollziehbar begründen lässt sich dies durch den vergleichsweise geringen Anteil an mathematischen Beiträgen in den wissenschaftsvermittelnden Druckmedien des 19. und 20. Jahrhunderts.

Der Befund, dass die Mathematik damit gleichsam „im Windschatten der Öffentlichkeit“ segelte (Nipperdey 1990: 605; Schwarz 1999: 105), spiegelt die besondere Entwicklung der Disziplin seit dem ausgehenden 19. Jahrhundert. Diese kennzeichnete etwa, dass sich mathematisches Wissen als unverzichtbare Ressource für zahlreiche Gebiete der Natur‑, Technik- und Sozialwissenschaften etablierte. Als Folge davon war deren disziplinäre Identitätsbildung begleitet von kontroversen Diskursen um das Für und Wider von Mathematisierung. Beispielhaft hierfür steht die sogenannte „Antimathematische Bewegung“ der 1890er Jahre (Hensel et al. 1989; Irrgang 2010: 140), in deren Rahmen sich eine Auseinandersetzung um eine für die Anwender*innen aus dem Bereich der Technik und der Ingenieurwissenschaften angemessene mathematische Ausbildung entspann. Dabei beklagten die Praktiker*innen die für ihre Anforderungen zu theoretisch geprägten mathematischen Lehrveranstaltungen. Gleichzeitig forderten die Mathematiker*innen die ihr disziplinäres Selbstbild im Verlauf des 19. Jahrhunderts mehr und mehr bestimmende Strenge ein.

Darüber hinaus setzten dieses Paradigma der Strenge und die zunehmend abstrakter werdenden Gegenstände aktueller mathematischer Forschung deren medialer Darstellung enge Grenzen. Die Deutungsmacht über ihre Inhalte zu sichern und sich in dem neu ausdifferenzierenden disziplinären Gefüge von Geistes- und Naturwissenschaften öffentlich zu positionieren, geriet für die Mathematik unter diesen Bedingungen zur Herausforderung.

Fragt man nun nach Genese und Zirkulation von bis in die Gegenwart wirksamen Selbst- und Fremdbildern einer Disziplin, die in hohem Maße für das theoretische Fundament des digitalen Zeitalters verantwortlich zeichnet (Mainzer 2018), ist es notwendig, Formen, Funktionen und Entstehungskontexte von Repräsentationen der Mathematik in den wissenschaftsvermittelnden Druckmedien des 19. und 20. Jahrhunderts zu analysieren.

Die der vorliegenden Fallstudie zugrunde liegende breit angelegte Untersuchung hatte sich deshalb der systematischen Auswertung einer umfangreichen Auswahl relevanter deutschsprachiger Zeitschriften- und Buchpublikationen^1^ für den Zeitraum von rund um 1880 bis 1960 angenommen. Dabei zeigte sich, dass die Präsenz der Mathematik im Wesentlichen folgenden drei mathematik- und wissenschaftshistorisch relevanten Kontexten zuordenbar ist:

1. der den Zeitraum von circa 1880 bis 1920 dominierenden Bildungsdiskussion und der damit einhergehenden pädagogischen Reformbewegung, in der die Mathematik insbesondere in der Person von Felix Klein (1849–1925), einem der führenden Mathematiker seiner Zeit und einflussreicher Wissenschaftsorganisator, eine zentrale Rolle einnahm,
2. der in den 1920er Jahren unter anderem im Kontext der Rezeption von Oswald Spenglers *Der Untergang des Abendlandes* aufkommenden Auseinandersetzungen um die Historizität von Wissenschaft im Allgemeinen und der Mathematik im Besonderen,
3. der über den gesamten Untersuchungszeitraum andauernden Kontroversen um die Anwendungen und die Anwendbarkeit der Mathematik.

Kennzeichnend für einen großen Teil der Beiträge war die Intention, über die Vermittlung mathematischen Wissens hinaus das „Wesen“ der Mathematik zu beschreiben. Damit fungierten die genannten Medien vor allem als Ort der Konstruktion identitätsbestimmender Selbstbilder zur Legitimierung der Mathematik und dienten kaum der Kommunikation schwer darstellbarer aktueller Forschungsergebnisse. Auch die sogenannte Unterhaltungsmathematik spielte nur eine untergeordnete Rolle.

Es ergab sich darüber hinaus, dass etwa 75 Prozent der für die Darstellungen verantwortlich zeichnenden Personen der Lehrerschaft mathematisch-naturwissenschaftlicher Fächer angehörte. Ihre überwiegende Mehrheit stand wiederum in engem Kontakt zu den akademischen Vertreter*innen der Disziplin, die sich zu einem vergleichsweise geringen Anteil direkt an diesen Aktivitäten beteiligten.^2^

**Figure 1:**
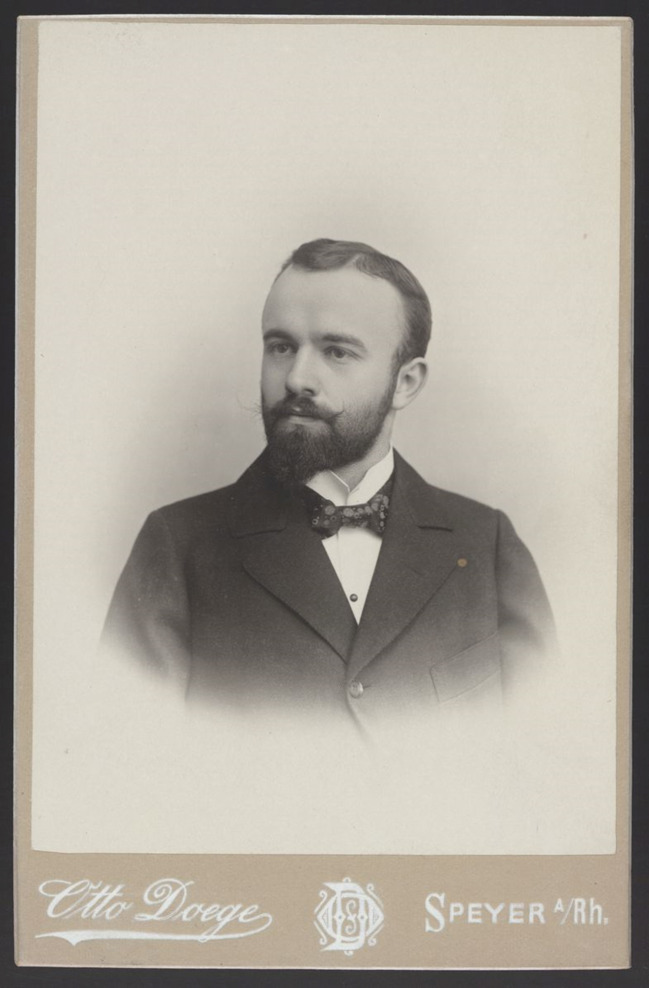

Einer von ihnen war der Pädagoge Heinrich Wieleitner (1874–1931) (Abb. 1), dessen umfangreiche Publikationstätigkeit im Kontext der hier im Vordergrund stehenden Beziehungen von Mathematikkommunikation und Mathematikgeschichtsschreibung einen ertragreichen Untersuchungsgegenstand darstellt. Bislang hat sich die wissenschaftshistorische Forschung ausschließlich auf Wieleitners Wirken als Mathematikhistoriker konzentriert (siehe etwa Dauben & Scriba 2002). Im Folgenden stehen deshalb die Verflechtungen von Wieleitners unterschiedlichen Betätigungsfeldern als Historiker, Kommunikator und auch als Pädagoge im Vordergrund.

Damit eröffnen sich unter Einbeziehung medienhistorischer Aspekte auch Einblicke in die personelle, institutionelle und inhaltliche Struktur mathematischer Öffentlichkeitsarbeit in den ersten Jahrzehnten des 20. Jahrhunderts. Der Fokus liegt dabei auf der Weimarer Zeit, deren kulturelle Debatten die Mathematik nicht nur auf besondere Weise herausforderten (Epple 2009; Carson et al. 2011; siehe auch den Abschnitt „Wieleitner und Mathematikkommunikation in der Weimarer Zeit“). Sie erwiesen sich, wie ich im Folgenden am Beispiel Wieleitners zeigen möchte, unter den Bedingungen einer sich verändernden Medienlandschaft auch als Chance für eine publizistisch erfolgreiche Selbstdarstellung der Disziplin.

## Heinrich Wieleitner: Lehrer, Historiker, Vermittler

Als 1911 Wieleitners erstes genuin mathematikhistorisches Werk erschien (Wieleitner 1911a), hatte er sich bereits als Autor mathematischer Lehrbücher und Aufsätze sowie als Verfasser von schulpolitischen Beiträgen etabliert.^3^ Auch veröffentlichte er seit 1903 in regelmäßigen Abständen in der Zeitschrift *Natur und Kultur* Beiträge zu mathematischen und naturwissenschaftlichen Themen (siehe beispielsweise Wieleitner 1903a, b, 1904, 1906). *Natur und Kultur*, gegründet 1903 von Franz Joseph Völler in München, war zeitweilig das Verbandsorgan der *Gesellschaft für Naturwissenschaften und Psychologie* und widmete sich von Beginn an einer liberalkatholisch orientierten breitenwirksamen Vermittlung der Naturwissenschaften (Daum 1998: 341, 372ff.).

Wieleitners über Jahrzehnte andauerndes Engagement an dieser Stelle erklärte sich unter anderem durch seine katholisch geprägte Herkunft und Erziehung. Aus einfachen Verhältnissen stammend besuchte er das bischöfliche Knabenseminar Scheyern und trat später in das Seminar in Freising ein. Noch vor dem Abitur fiel jedoch die Entscheidung gegen die Laufbahn als Geistlicher, und Wieleitner begann 1893 ein Studium der Mathematik an der Universität München, das er zunächst größtenteils durch Nachhilfeunterricht finanzierte. 1895 bestand er das Zwischenexamen und erhielt danach auf Vorschlag seines späteren Doktorvaters Ferdinand Lindemann (1852–1939) Unterstützung durch das sogenannte große Lamont-Stipendium (um diese Förderung zu erhalten, musste man unter anderem katholisch und in Bayern geboren sein).

Nach abgeschlossenem Examen und einer kurzzeitigen Beschäftigung als Hilfsassistent des Mathematikers Walther von Dyck (1856–1934) an der Technischen Hochschule München trat Wieleitner zu Beginn des Jahres 1900 in den Schuldienst ein. Er begann seine Lehrerlaufbahn in Speyer, wo er 1901 seine Dissertation *Über die Flächen dritter Ordnung mit Ovalpunkten* (Wieleitner 1901) fertigstellte. Weitere Stationen waren Pirmasens und Augsburg. 1926 kehrte er nach München zurück und war dort bis zu seinem Tod als Rektor und Oberstudiendirektor am Neuen Realgymnasium tätig.

Wieleitner pflegte einen großen Bekannten- und Freundeskreis, der organisch mit seinem nationalen und internationalen professionellen Netzwerk verknüpft war. Davon zeugt die umfangreiche Korrespondenz in seinem seit einiger Zeit im Archiv des Deutschen Museums zur Verfügung stehenden Nachlass.^4^

Eine über Jahrzehnte währende Bekanntschaft verband ihn mit Siegmund Günther (1848–1923), der seit 1874 zunächst als Lehrer für Mathematik und Physik tätig war und danach von 1886–1920 an der TH München als Professor die Geografie und die Geschichte der Naturwissenschaften vertrat. Günthers Bibliografie umfasste neben Veröffentlichungen zu mathematischen Forschungsfragen Beiträge zur Meteorologie, zur Geophysik und zur Geschichte der Mathematik und der Naturwissenschaften. Er war auch Autor einer Reihe von nichtakademische Leserkreise adressierenden einführenden Lehrbüchern, betreute als Herausgeber die allgemeinbildende Buchreihe *Bücher der Naturwissenschaft* (Reclam, Leipzig) und betätigte sich als Berichterstatter für die populärwissenschaftliche Zeitschrift *Die Natur* (Daum 1998: 327, 489). Günther gehörte auch gemeinsam mit dem seit 1892 an der Technischen Hochschule München lehrenden Mathematikhistoriker Anton von Braunmühl (1853–1908) seit den 1880er Jahren zu den führenden Protagonisten bei den Bestrebungen, die Geschichte der Mathematik als Lehrfach an den Universitäten zu etablieren (Dauben & Scriba 2002: 125–131).

1908 veröffentlichte Günther in der beim Verlag Göschen herausgegebenen mathematischen Lehrbuchreihe *Sammlung Schubert* (benannt nach dem Herausgeber, dem Mathematiker Hermann Schubert [1848–1911]) den ersten Band einer zweibändig angelegten und bereits 1902 angekündigten *Geschichte der Mathematik* (Günther 1908). Sie war einerseits als Übersichtsdarstellung und damit als Ergänzung und Aktualisierung des mehrbändigen und umfangreichen Standardwerkes des Mathematikhistorikers Moritz Cantor (1829–1920) konzipiert (Cantor 1908). Gleichzeitig sollte sie in der Ausführung den Ansprüchen der *Sammlung Schubert* genügen, die die „fassliche Darstellung“ mit „wissenschaftlicher Strenge“ verbunden sehen wollte und genau aus diesem Grunde bei Fachmathematiker*innen ein hohes Ansehen genoss (Remenyi 2008: 87, 106).

Für den zweiten Band hatte man als Autor Anton von Braunmühl verpflichtet, dessen mit zahlreichen Anmerkungen versehenes Manuskript nach seinem Tod eines/r sachkundigen Bearbeiters/*in bedurfte. Wieleitner war zu diesem Zeitpunkt bereits mit zwei Lehrbüchern zum Themenkomplex der algebraischen Kurven (Wieleitner 1905, 1908a) als Autor der *Sammlung Schubert* etabliert. Kennzeichnend für diese Texte war nicht nur eine für Anfänger*innen gut lesbare Aufbereitung des Materials. Sie zeigten auch Wieleitners Interesse an einer historischen Einordnung mathematischer Ergebnisse, nicht zuletzt zum Zwecke einer schlüssigen und leicht nachvollziehbaren Darstellung abstrakter Begriffsbildungen. Dieses Interesse mag auch durch entsprechende Aktivitäten seiner Mentoren Walther von Dyck und Ferdinand Lindemann begründet gewesen sein.^5^ So war es wenig erstaunlich, dass Siegmund Günther im Einvernehmen mit Verlag und Herausgeber Heinrich Wieleitner 1908 die Fertigstellung des zweiten Bandes der *Geschichte der Mathematik* auf Basis von von Braunmühls Unterlagen anvertraute. Inhaltlich widmete sich der Band, dessen erster Teil 1911 (Wieleitner 1911a) und dessen zweiter Teil 1921 (Wieleitner 1921b) erschien, dem Zeitraum vom Wirken Descartes’ bis zur Wende des 18. Jahrhunderts.

Seit Beginn seiner Tätigkeit als Pädagoge war Wieleitner auch schulpolitisch aktiv. So publizierte er 1908 in einer Beilage der *Münchner Neuesten Nachrichten* einen Beitrag zum mathematischen Unterricht an den Mittelschulen (Wieleitner 1908b), der die Aufmerksamkeit von Julius Ruska (1867–1949) auf sich zog (Ruska 1932). Ruska, zunächst Lehrer für Mathematik und Naturwissenschaft, später Orientalist und als Wissenschaftshistoriker Direktor des 1927 gegründeten Forschungsinstituts für die Geschichte der Naturwissenschaften (Universität Berlin), war im Zeitraum von 1908 bis 1913 Herausgeber der Zeitschrift *Das Pädagogische Archiv. *Es gelang ihm, Wieleitner, in dem er einen „Gleichgesinnten“ für „die Schulkämpfe der Zeit“ erkannte (Ruska 1932: 151), als Mitarbeiter für diese *Monatsschrift für Erziehung, Unterricht und Wissenschaft*, so der Untertitel, zu gewinnen. In dem 1910 dort veröffentlichten Aufsatz *Bayerische Schulfragen* (Wieleitner 1910) bestätigte Wieleitner seine positive Haltung zu den Inhalten der Meraner Schulreform (Schubring 2007). Deren Forderungen umfassten neben der stärkeren Berücksichtigung der Anschauung im Mathematikunterricht auch die Schärfung des funktionalen Denkens durch Einführung der Differenzial- und Integralrechnung in den Lehrplan der höheren Schulen.

Wieleitner betonte darüber hinaus die Fruchtbarkeit der konsequenten Einübung mathematischer Techniken und plädierte für ein ausgewogenes Verhältnis von logischer Strenge und Intuition im mathematischen Unterricht sowohl an den höheren Schulen als auch bei den einführenden Veranstaltungen der Universitäten (Wieleitner 1910: 367ff.).

Die stetige und aktive Auseinandersetzung mit mathematischen Methoden erschien Wieleitner gleichermaßen als probates Mittel, um dem Akzeptanzproblem der Mathematik in den sogenannten breiteren Kreisen zu begegnen. Dazu betreute er seit 1909 bis zu seinem Tode bei *Natur und Kultur* eine Rubrik, die in jedem Heft eine Aufgabe aus dem Bereich der Elementarmathematik präsentierte, häufig versehen mit einem historischen Kontext und regelmäßig begleitet von dem Aufruf, Lösungen einzusenden. In der Folgeausgabe wurden zusätzlich zur Darstellung von korrekten Lösungswegen auch deren Einsender*innen namentlich mit Berufsbezeichnung genannt, was einen einzigartigen Einblick in die professionelle Verortung der Rezipient*innen ermöglicht. Neben den zu erwartenden Vertreter*innen der Lehrerschaft und des geistlichen Standes, Schüler*innen und Studierenden trifft man dabei gleichermaßen auf Praktiker*innen, etwa aus dem Bereich der Architektur oder der Forstwirtschaft, wie auf Angehörige der Arbeiterschaft.

Wieleitners in *Natur und Kultur *veröffentlichte Aufsätze kennzeichnete neben ihrer thematischen Vielfalt auch eine enge Verflechtung mit seiner mathematikhistorischen Forschungstätigkeit. Als Folge der Vorstudien zum zweiten Teil der *Geschichte der Mathematik* war von 1911–1920 eine Reihe von Veröffentlichungen entstanden, die sich insbesondere der Geschichte der grafischen Darstellung und der Behandlung des Fallgesetzes in der Spätscholastik widmeten. Publikationsorte waren dabei die mathematikhistorische Fachzeitschrift *Bibliotheca Mathematica* (Wieleitner 1912a) und die *Zeitschrift für den mathematischen und naturwissenschaftlichen Unterricht *(Hofmann 1933: 215ff.). Die Beiträge, welche die entsprechenden Ergebnisse für ein Laienpublikum aufbereiteten, erschienen neben *Natur und Kultur* auch in *Das Weltall *(UT: *Illustrierte Zeitschrift für Astronomie und verwandte Gebiete*) (Wieleitner 1916a, Wieleitner 1916b).

Beispielhaft zeigten Wieleitners Untersuchungen zur grafischen Darstellung seine Auffassung von aus seiner Sicht angemessener mathematikhistorischer Methodik, deren Kern die präzise philologische Interpretation von Primärquellen konstituierte (Wieleitner 1912a: 116). Dazu bedurfte es fundierter Sprachkenntnisse, umfassender bibliografischer Recherchen und der Beschaffung entsprechender Literatur. Wieleitners Expertise in diesem Zusammenhang entsprang neben seiner Vielsprachigkeit einer über Jahrzehnte dauernden umfangreichen Rezensionstätigkeit. Er verfasste viele hundert Buchbesprechungen und Zusammenfassungen von Zeitschriftenaufsätzen unter anderem im *Pädagogischen Archiv*, im *Archiv der Mathematik und Physik *und der* Deutschen Literaturzeitung*. Nach dem Tod Siegmund Günthers 1923 übernahm er die Redaktion für Mathematik und Naturwissenschaften bei den *Mitteilungen zur Geschichte der Medizin und der Naturwissenschaften*, dem seit 1901 erscheinenden Organ der im gleichen Jahr gegründeten *Deutschen Gesellschaft für Geschichte der Medizin und der Naturwissenschaften*. Wieleitner sorgte damit in den 1920er Jahren durch eine Vielzahl von Beiträgen für eine ungewöhnlich hohe Präsenz mathematischer Themen in diesem zentralen Kommunikationsmittel der deutschsprachigen Wissenschaftsgeschichte. Da seine mathematikhistorischen Publikationen ihm seit 1911 Zug um Zug auch zu internationaler Reputation verholfen hatten, datierte der Beginn seiner Mitarbeit bei *Isis* und dem *Archivio di Storia della Scienzia *ebenfalls in die beginnenden 1920er Jahre.

Die Forschungsarbeit seines letzten Lebensjahrzehnts war bestimmt durch die Hinwendung zur Geschichte der indischen, ägyptischen und arabischen Mathematik. Von einem diesbezüglichen internationalen wissenschaftlichen Austausch zeugt etwa die Korrespondenz mit den indischen Mathematikhistoriker Bibhūtibhūṣaṇ Datta (1888–1958). Wieleitners Anfrage nach Beziehungen zwischen indischer und griechischer Mathematik – die von Datta abschlägig beschieden wurde –6 wies dabei auf seine Bestrebungen hin, eine umfassende und sich auf epistemische Kontinuitäten stützende Ideengeschichte der Mathematik zu konzipieren. Gleiches lässt sich aus der Korrespondenz mit dem Ägyptologen Ludwig Borchardt (1863–1938) im Zusammenhang mit Wieleitners Aufsatz *War die Wissenschaft der alten Ägypter wirklich nur praktisch?* (Wieleitner 1927) schließen. Borchardt widersprach dort der These Wieleitners, nach der die ägyptische Wissenschaft und damit insbesondere die Mathematik von den Altphilologen zu Unrecht und aus Mangel an wissenschaftshistorischen und mathematischen Kenntnissen als rein empirisch eingeordnet und damit mit einem „Unterton des Herabsetzenden“ (Wieleitner 1927: 13) beschrieben worden sei.^7^

Wieleitners nichtfachwissenschaftliche Publikationstätigkeit dieser Jahre spiegelte diese Haltung, indem sie eine Reihe von Überblicksdarstellungen aufweist, die sich gleichermaßen zum Ziel setzten, innere Zusammenhänge zwischen moderner, zeitgenössischer und alter Mathematik zu benennen sowie mathematische und wissenschaftshistorische Kompetenzen zu vermitteln. Genannt seien hier zum einen die kürzeren Beiträge *Zur Erfindung der Infinitesimalrechnung* und *Zur Erfindung der analytischen Geometrie* in der von Wilhelm Dieck (1867–1935) herausgegebenen mehrbändigen Aufsatzsammlung *Mathematisches Lesebuch*, die sich unter anderem als Begleitlektüre zu Volkhochschulkursen verstand (Wieleitner 1920, 1921a). Demgemäß stand dort die Schilderung historischen Grundlagenwissens verbunden mit einer elementaren Einführung in die jeweiligen mathematischen Theorien im Vordergrund.

Die in der fächerübergreifenden *Sammlung Göschen* erschienenen Bände *Geschichte der Mathematik* (Wieleitner 1922, 1923) hatten dem Profil der Reihe (Remenyi 2008: 88) entsprechend den Charakter „kurz gefasster, allgemeinverständlicher Einzeldarstellungen“. Zugunsten der kulturhistorischen Einordnung der Mathematik von den „ältesten Zeiten“ (so der Untertitel des ersten Bandes) bis zur Mitte des 19. Jahrhunderts nahm die Vermittlung von mathematischen Techniken vergleichsweise wenig Raum ein.

Zu der mathematisch-physikalischen Themen vorbehaltenen, allgemeinbildenden Reihe* Mathematisch-Physikalische Bibliothek*, herausgegeben von den Mathematiklehrern Walther Lietzmann (1880–1959) und Alexander Witting (1861–1946) (Remenyi 2008: 93 f.), hatte Wieleitner bereits 1911 und 1912 mit je einer Darstellung zum Zahlbegriff und zur elementaren Arithmetik beigetragen (Wieleitner 1911b, 1912b). 1925 erschien dort der Band *Der Gegenstand der Mathematik im Lichte ihrer Entwicklung* (Wieleitner 1925a), der sich – bezogen auf die Zielsetzung, einem Laienpublikum in erster Linie das Wesen und damit die Relevanz der Mathematik nahe zu bringen – in eine ganze Reihe von Publikationen anderer Autor*innen dieser Zeit mit vergleichbarer Intention einordnen lässt (siehe etwa Heffter 1922; Voss 1908).

## Autobiografie einer Disziplin: *Die Geburt der modernen Mathematik*

Der Charakterisierung disziplinärer Identität widmete sich auch das zweibändige Werk *Die Geburt der modernen Mathematik* (Wieleitner 1924b, 1925b). Die beiden jeweils etwa siebzig Seiten umfassenden Teile erschienen in der Reihe *Wissen und Wirken, Einzelschriften zu den Grundlagen des Erkennens*, die sich nicht an „jedermann“ richtete, sondern „neues Wissen“ für ein akademisch vorgebildetes Publikum präsentierte, so ihr Anspruch. Gleichwohl erwartete man von den Autor*innen eine „schlichte Sprache“ und setzte sich zum Ziel, einen Beitrag „zur Verständigung über Ziele und Wege der zeitgenössischen Kultur zu leisten“^8^.

Wieleitner nutzte diesen Publikationsort, um anhand der Geschichte der analytischen Geometrie (Band I) und der Infinitesimalrechnung (Band II) einem akademisch vorgebildeten Publikum sowohl die kulturelle Verankerung und Bedeutung der Mathematik als auch deren Rolle als disziplinübergreifende Ressource zu vermitteln.

Ausgehend von der Verortung der „Geburt“ der modernen Mathematik im 17. Jahrhundert, betonte Wieleitner eingangs die Relevanz der analytischen Geometrie für die Entstehung und Entwicklung der modernen Wissenschaft. Daraus leitete er ab, dass ein tiefergehendes Verständnis gegenwärtiger und vergangener naturwissenschaftlicher Errungenschaften und damit der „modernen Kultur“ (Wieleitner 1924b: 3, 4) nur über eine ausreichende Kenntnis entsprechender mathematischer Grundlagen möglich sei: „Es ist überall letzten Endes die Mathematik, die mangelt“.^9^ Aufbauend auf dieser Legitimierung sowohl der Mathematik als auch der Wissenschaftsgeschichte entfaltete Wieleitner ein mathematikhistorisches Panorama, das, beginnend bei der griechischen Mathematik, über die Entwicklung der Algebra bis zu den Beiträgen von Pierre de Fermat und René Descartes zur analytischen Geometrie im 17. Jahrhundert führte.

Der zweite Band widmete sich zunächst einem Exkurs über das Unendliche, wiederum rekurrierend auf die Wurzeln der Begriffsbildung bei den Griechen. Deren Methoden zur Flächen- und Volumenberechnung gemeinsam mit einem an Beispielen erläuterten Grenzwertbegriff aus der zweiten Hälfte des 19. Jahrhunderts ermöglichten Wieleitner im weiteren Verlauf die Präsentation der Grundlagen der Integralrechnung.

Die in Teil I bereitgestellten Erkenntnisse zur Entwicklung der analytischen Geometrie führten weiter zu den Problemstellungen der Differenzialrechnung (Gottfried Wilhelm Leibniz und Issac Newton) und damit insbesondere zur Darstellung des erstmals von Isaac Barrow (1630–1677) bewiesenen Hauptsatzes der Differenzial- und Integralrechnung. Damit war der Boden bereitet, um in Grundzügen ein mathematisches Konzept vorzustellen, das nach Wieleitner die Basis für jede tiefenscharfe Analyse von Naturphänomenen bildete: die Differenzialgleichung.

Im Kontext dieser Erzählung verfolgte der Text im Wesentlichen drei Ziele:

1. Formulierung von Argumenten sowohl für die Kontinuität als auch die Historizität der disziplinären Entwicklung der Mathematik,
2. Darstellung wesentlicher Konstituenten mathematischer Forschungspraxis,
3. Inszenierung der Mathematik als Grundlage für die exakten Naturwissenschaften und damit für alle Formen technisch-zivilisatorischer Entwicklung und als wesentliche Wissensressource zum Verständnis zentraler philosophischer Fragestellungen.

### Zu 1.:

Neben der wiederholten Feststellung, dass die „moderne Mathematik einer Wiedererweckung und Fortbildung der Mathematik der Alten zu danken“ sei (Wieleitner 1924b: 14 f.), verwies Wieleitner gleichermaßen auf Kontinuitäten zwischen den dargestellten Entwicklungen des 17. Jahrhunderts und mathematischen Konzepten des 19. und 20. Jahrhunderts (Wieleitner 1924b: 56 f.). Das schlagkräftigste Beispiel in diesem Zusammenhang bezog sich dabei auf die Koordinatengeometrie als Grundlage für die Entwicklung einer Geometrie von mehr als drei Dimensionen.

Gleichwohl betonte er in einem deutlich höheren Maß als in anderen Veröffentlichungen, dass sowohl mathematische Gegenstände als auch mathematische Denkweisen historischer Veränderlichkeit unterliegen. Insbesondere bezog er sich dabei auf die begriffliche Strenge der griechischen Mathematik, die mit dem einsetzenden Mittelalter zunächst über Jahrhunderte an Bedeutung verloren habe. Erst im 19. Jahrhundert sei die „ungeheuer entfaltete Mathematik in ihren Hauptteilen wieder zur griechischen Strenge zurückgebracht“ worden (Wieleitner 1925b: 2). Diese Form der dynamischen Entwicklung epistemischer Paradigmen benannte Wieleitner als unabdingbar für die konstruktive Weiterentwicklung mathematischer Erkenntnis.

### Zu 2.:

Mathematische Arbeit charakterisierte Wieleitner durch die Verflechtung folgender Praxen: Lösung von Einzelproblemen, systematische Entwicklung von Methoden, Formulierung von Verallgemeinerungen. Um über diesen Prozess sowohl kurzfristig als auch in der historischen Perspektive zu neuen Einsichten zu gelangen, konstatierte er die Notwendigkeit adäquater Bezeichnungen und Begriffsbildungen. Beispielhaft formulierte er dies im historischen Kontext in der folgenden Passage aus Band I:

„Für den Griechen war jede Aufgabe ein neues Problem. […] Der Begriff der ‚Methode‘ fehlte fast ganz. Nun ist ja freilich die eigentliche Elementarmathematik zur Anwendung einer einheitlichen Methode nicht geeignet. Aber gerade bei den Örtern, d. h. in der Behandlung der Linien ersten und zweiten Grades, wie wir sagen, konnte Fermat deutlich zeigen, welch großen Wert ein methodisches Verfahren hat. Daß die Griechen diese Verfahren nicht hätten anwenden können, auch wenn sie den Sinn dafür gehabt hätten, wird nach den bisherigen Erläuterungen wohl deutlich. Es fehlte ihnen die Buchstabenalgebra.“ (Wieleitner 1924b: 37)

Die analytische Geometrie als „in Algebra übersetzte Geometrie“ (Wieleitner 1924a:171) beschrieb Wieleitner als Grundlage für die Entstehung vielfältiger konzeptioneller Verallgemeinerungen (Wieleitner 1924b: 53 ff.) der Mathematik des 19. und 20. Jahrhunderts und benannte insbesondere deren essenzielle Bedeutung für die Entwicklung der Differenzialrechnung: „Descartes hatte ja gezeigt, daß jede algebraische Gleichung zwischen den Koordinaten x, y eine Kurve darstellt (vgl. Bd. I, S. 7 f.). Von der Untersuchung dieser Kurven und der damit zusammenhängenden algebraischen Gleichungen ging der Anstoß zur Erfindung der Differentialmethode aus“ (Wieleitner 1925b: 34 f.).

### Zu 3.:

Ausgehend von der Beobachtung, dass die auf ebendieser Differenzialmethode beruhende Aufstellung und Lösung von Differenzialgleichungen als schlagkräftige Methode zum Verständnis von Naturerscheinungen etabliert und anerkannt sei, stellte Wieleitner weiter fest: „Die gesamte exakte Naturwissenschaft beruht daher auf der Infinitesimalrechnung“ (Wieleitner 1925b: 63). Die essenzielle Bedeutung der Mathematik nicht nur für die technisch-zivilisatorische Entwicklung, sondern auch für das von Nützlichkeitserwägungen befreite Suchen nach einem tieferen Verständnis der Natur war bereits zu Beginn von Band I ausführlich erläutert worden (Wieleitner 1924b: 2 f.).

Wieleitner ging in seiner Argumentation zum Ende des zweiten Bandes noch einen Schritt weiter und benannte mathematisches Wissen als wesentliche Kompetenz für eine angemessene Interpretation der cartesischen und der leibnizschen Philosophie, wobei er insbesondere Gottfried Wilhelm Leibniz’ Beitrag zur Lösung des Leib-Seele-Problems durch das Konzept der prästabilierten Harmonie als Differenzialgesetz paraphrasierte:

„Leibniz’ Gedanke ist, modern ausgedrückt, der, daß Leib und Seele in funktionaler Beziehung zu einander stehen. Nur der mathematisch Gebildete aber wird die Tiefe dieses Gedankens ganz erfassen und die Oberflächlichkeit jeder anderen Erläuterung der prästabilierten Harmonie, (wie der von den zwei synchron laufenden Uhren), scharf durchschauen.“ (Wieleitner 1925b: 64 f.).

*Die Geburt der modernen Mathematik *bot so, ganz im Sinne des Untertitels *Historisches und Grundsätzliches*, nicht nur einen historischen Überblick zur Entwicklung der analytischen Geometrie und der Infinitesimalrechnung. Über die Verbreitung mathematischer und historischer Fakten hinaus versuchte sich die Publikation in einer Charakterisierung der Mathematik sowohl bezüglich ihrer Eigenschaften als autonome Disziplin als auch ihren Beziehungen zu Zivilisation und Kultur. Mathematischem Wissen kam dabei sogar die Rolle des positiven Vermittlers zwischen diesen seit der Wende zum 20. Jahrhundert vielfach als gegensätzlich wahrgenommenen Begriffen zu, womit es besonders geeignet schien, die von den Herausgebern der Reihe beschworene „seelische Not unserer Zeit“ (Wieleitner 1924b: I) zu lindern. Wieleitner präsentierte damit in gewisser Weise eine „Autobiographie“ (Jurdant 1993)^10^ der Mathematik, die sowohl der Legitimierung jenseits der Fachwissenschaft als auch der Selbstverständigung und Selbstvergewisserung innerhalb der Disziplin dienen konnte. Die sprachliche Realisierung der Darstellung kennzeichnete dabei die häufige Verwendung lebensweltlicher Begriffe^11^ und eine Personalisierung der Erzählung durch ausführliche biografische Exkurse zu den wichtigsten Protagonisten Descartes, Fermat, Leibniz und Newton. Darüber hinaus diente eine Reihe von Illustrationen der Veranschaulichung mathematischer Inhalte. Die Integration der Formelsprache berücksichtigte die Bedürfnisse eines der Mathematik nur bedingt kundigen Publikums. Gleichzeitig bemühte sich der Text um einen sorgfältigen Umgang mit einem zentralen erkenntnistheoretischen Begriff der Mathematik: dem Beweis. Überall dort, wo die Komplexität der mathematischen Argumentation bei der Vermittlung Beschränkungen in der Exaktheit auferlegte, setzte Wieleitner diesen Begriff in doppelte Anführungszeichen und wurde so auch einem mathematisch gebildeten Publikum gerecht.

## Wieleitner und die mathematische *Scientific Community*

Beide Bände von *Geburt der modernen Mathematik *erfuhren eine breite positive Rezeption. Der Verlagskorrespondenz ist zu entnehmen, dass „sie mit am besten von den 60 Bändchen der Sammlung gingen“.^12^ Dabei umfasste die Reihe so prominente Autoren wie Gertrud Bäumer, Rudolf Carnap und Hans Driesch, die sich zum Teil öffentlichkeitswirksameren Themen – wie etwa der Relativitätstheorie – widmeten.

Zustimmende Reaktionen gab es auch aus den Reihen der Hochschulmathematiker. So schrieb Otto Toeplitz (1881–1940) im Juni 1925 an Wieleitner:

„Die ‚Geburt der modernen Mathematik‘ habe ich mir sofort zu beschaffen gesucht, und sie ist soeben in meine Hände gelangt, und ich habe Bd. 2 rasch gelesen. Ich freue mich, die Grundauffassung auch von Ihnen verfochten zu wissen, daß der Unterschied von heutiger und griechischer Mathematik oft übertrieben wird und innerlich auf ein Geringes zusammenschrumpft.

Der Aufsatz, in dem ich das für die ‚Antike‘, also ein sehr breit zu nehmendes Publikum mit den mir wesentlichen Argumenten darzulegen versucht habe, ist die Ausarbeitung eines Vortrages über Spengler, den ich in der hiesigen Ortsgruppe der Kantgesellschaft im Sommer 1920 gehalten habe, und die damals einer Reihe von Fachgenossen vorgelegen hat.“^13^

Toeplitz’ Kommentar ordnete sich ein in den Kontext der Reaktionen von Fachmathematiker*innen auf Oswald Spenglers Werk *Der Untergang des Abendlandes*. Die herausragende Rolle der Mathematik in dieser kulturpessimistischen Darstellung des Spannungsfeldes zwischen Zivilisation und Kultur verschaffte der Disziplin einerseits eine durchaus erwünschte Aufmerksamkeit, indem ihr eine tiefgehende kulturelle Bedeutung zugestanden wurde. Spenglers Beschreibung der Historizität mathematischer Praxis implizierte jedoch gleichermaßen den Mangel an kontinuierlicher disziplinärer Entwicklung und stellte darüber hinaus die zeitgenössische, moderne Mathematik in Frage (Epple 2009: 145ff.). Toeplitz’ Anliegen in dem von ihm erwähnten Aufsatz (Toeplitz 1925) war es, mit explizitem Bezug auf Spengler (ebd. 1925: 177) durch eine tiefgehende Auseinandersetzung mit der Geschichte der Mathematik „Kontinuität *und* historische Veränderung mathematischer Arbeit erkennbar“ (Epple 2009: 147) zu machen. Die moderne Mathematik sollte so als Produkt einer dynamischen Weiterentwicklung von Methoden entlang von Fragestellungen gekennzeichnet werden, die bereits in der Antike im Mittelpunkt mathematischer Praxis standen. Damit deckten sich seine Auffassungen in weiten Teilen mit den von Wieleitner entwickelten Gedanken, wenngleich dieser sich in seinen Ausführungen nicht auf Spengler bezog. Eine weitere Gemeinsamkeit zwischen Toeplitz’ und Wieleitners Ausführungen bestand darin, dass beide die Schilderung mathematischer Arbeit gegenüber der Beschreibung mathematischer Gegenstände in den Vordergrund rückten.

Die Korrespondenz zu diesem Themenkomplex bildete den Ausganspunkt für einen bis zu Wieleitners Tod währenden wissenschaftlichen und persönlichen Austausch.

So gratulierte Toeplitz im April 1928^14^ zu Wieleitners Habilitation an der Universität München für Geschichte der Mathematik und setzte sich dabei eingehend mit den sechs Thesen des Probevortrags auseinander. Neben Äußerungen zur historischen Einordnung von Leibniz’ Beiträgen zur Erfindung der Differenzialrechnung und dem Wesen der ägyptischen Mathematik (Thesen 4 und 5) plädierte Wieleitner zum einen für die Anerkennung der Geschichte der Wissenschaften als „Bestandteil der Geschichte der menschlichen Kultur“ und bekannte sich dezidiert zu einer autonomen Entwicklung der reinen Mathematik: „Die Forschung und Entwicklung in der reinen Mathematik muß völlig ungehindert durch philosophische und praktische Rücksichten erfolgen.“ (Hofmann 1933: 211, Thesen 1und 3). These 2 stellte die Bedeutung der Geschichte der Wissenschaften für die Ausbildung von Mathematiklehrer*innen in den Vordergrund, während These 6 folgende Forderung formulierte: „Der Lehrer der Mathematik sollte immer in enger Fühlung mit der lebendigen Wissenschaft bleiben.“ (Hofmann 1933: 211) Toeplitz stimmte Wieleitner in oben genanntem Schreiben in denjenigen Positionen uneingeschränkt zu, die die Lehrerausbildung, die Autonomie der reinen Mathematik und die Relevanz der Geschichte der Wissenschaften betrafen. Die genuin mathematikhistorischen Thesen teilte er hingegen nur bedingt. Dies ist beispielhaft für die divergierenden Auffassungen von Toeplitz und anderen in der Geschichte der Mathematik engagierten Hochschulmathematiker*innen im Vergleich zu den der Lehrerschaft angehörenden Mathematikhistorikern wie Wieleitner und Johannes Tropfke (1866–1939) (Dauben & Scriba 2002: 135ff.). Während Letztere eine auf Primärquellen basierende Darstellung der Geschichte der Mathematik als Teil der allgemeinen Wissenschafts- und damit Kulturgeschichte in den Vordergrund stellten, war etwa für Toeplitz „das Arbeitsgebiet der Historie“ vor allem „ein Hilfsmittel für didaktische Zwecke“^15^. Im Sinne der sogenannten „generischen Methode“ war es sein Anliegen, „aus der Historie nur die Dinge, die sich bewährt haben herauszugreifen, und sie direkt oder indirekt verwerten“, um aufzuzeigen, was an der Mathematik „spannend“ und „schön“ ist (Toeplitz 1927: 92, 94, 90).

Trotz dieser methodischen Differenzen und der Einschätzung, dass Wieleitners und Tropfkes „Niveau seine Grenzen“^16^ habe, bewertete Toeplitz Wieleitners Tätigkeit als Privatdozent für Geschichte der Mathematik an der Münchener Universität uneingeschränkt positiv. Er sah darin die Chance, Studierende für die Historiografie der Mathematik zu gewinnen und damit zu deren Etablierung und Professionalisierung beizutragen.^17^

Eine derartige Entwicklung erschien nicht zuletzt deshalb erstrebenswert, weil mathematische Geschichtsschreibung in den 1920er Jahren jenseits einer Studierende adressierenden didaktischen Methode für die Hochschulmathematiker*innen eine weitere wichtige Funktion erfüllte. Sie erlaubte eine Form der erzählenden disziplinären Selbstbeschreibung und Legitimierung, die besonders dazu geeignet war, die Mathematik als sowohl für philosophische als auch naturwissenschaftliche Fragen relevant und somit als kulturell integrierende Kraft zu präsentieren. Von diesen Bestrebungen zeugen beispielweise eine Reihe von Beiträgen herausragender Mathematiker*innen seit der Mitte der 1920er Jahre in der Zeitschrift *Die Naturwissenschaften* (Schirmeier 2008).

In diesem Kontext erwies sich Wieleitner aufgrund seiner den Ansprüchen von Fachmathematiker*innen genügenden Art der Darstellung und seiner Auffassung vom Stellenwert und Charakter der Disziplin als wertvoller Verbündeter. Denn im Rahmen seiner umfangreichen Publikationstätigkeit erreichte er eine Vielzahl unterschiedlicher Rezipient*innen weit jenseits der Reichweite eines Organs wie *Die Naturwissenschaften*, das sich mit einer Auflage von etwa 2.000–3.000 Exemplaren in erster Linie an Wissenschaftler*innen anderer Fachgebiete richtete. Diese Tätigkeit erfuhr ihre Würdigung seitens der Disziplin nicht nur über eine Honorarprofessur an der Universität München, Wieleitner wurde 1930 auch in den Ausschuss und damit das Führungsgremium der Deutschen Mathematiker-Vereinigung gewählt.^18^

## Wieleitners Erbe

Während seiner – als Folge seines frühen Todes – recht kurzen akademischen Lehrtätigkeit hatte Wieleitner einige Schüler, von denen mit Kurt Vogel (1888–1985) und Joseph Ehrenfried Hofmann (1900–1973) mindestens zwei für die Entwicklung der Mathematikgeschichte als wissenschaftliche Disziplin in Deutschland vor und nach 1945 eine wichtige Rolle spielten.

Vogel hatte nach einem Studium der Mathematik und Physik in Erlangen und Göttingen bei Oskar Perron und Heinrich Wieleitner zu einem historischen Thema im Kontext des Papyrus Rhind 1929 in München promoviert. Er beschäftige sich in den folgenden Jahren unter anderem mit babylonischer Mathematik und deutschen Rechenbüchern des 15. und 16. Jahrhunderts und war neben seiner Tätigkeit als Lehrer seit 1940 auch als außerplanmäßiger Professor an der Universität München tätig. 1963 war er wesentlich an der Gründung des Instituts für Geschichte der Naturwissenschaften und Mathematik in München beteiligt. Der Vorläufer dieses Instituts war eine entsprechende Abteilung am Mathematischen Institut der Universität, deren Bibliothek aus Wieleitners ursprünglicher Privatbibliothek aufgebaut wurde (Dauben & Scriba 2002: 555ff.).

Recherchen im Bundesarchiv Koblenz und Archiv der LMU München haben keine Hinweise darauf ergeben, dass Vogel in der Zeit des Nationalsozialismus Mitglied einer der Gliederungen des NS war. Allerdings verweisen einige seiner Publikationen (beispielsweise: Die mathematischen Leistungen der Semiten im Altertum und Mittelalter. *Zeitschrift für die gesamte Naturwissenschaft* 5 [1939]: 88–93 [in der Abt. Berichte und Mitteilungen]; Mathematik und Judentum. *Zeitschrift für die gesamte Naturwissenschaft* 5 [1939]: 27 f) in den 1930er Jahren auf nationalsozialistisches Gedankengut.

Hofmann hatte 1927 bei Walther von Dyck und Georg Faber (1877–1966) zu einem Thema aus der reinen Mathematik promoviert und war dort bereits mit mathematikhistorischer Arbeit in Berührung gekommen. Die endgültige Zuwendung zur Mathematikgeschichte vollzog sich während seiner Tätigkeit als Mitarbeiter von Heinrich Wieleitner (die er neben seiner Anstellung im Schuldienst ausführte), der aufgrund einer schweren chronischen Erkrankung in seinen letzten Lebensjahren auf kundige Unterstützung angewiesen war. Nach Wieleitners Tod übernahm Hofmann nicht nur Wieleitners mathematikhistorisches Konzept einer Ideengeschichte, das er in den folgenden Jahren zu einer sogenannten Problemgeschichte modifizierte. Er „erbte“ auch dessen Kontakte zu in- und ausländischen Wissenschaftshistorikern (zu den genannten biografischen Angaben siehe etwa Folkerts 2011) und er trat in zahlreichen Medien Wieleitners Nachfolge als Kommunikator an. Neben der Arbeit für einige wichtige Referateorgane (siehe etwa *Jahrbuch für Fortschritte der Mathematik*) führte er Wieleitners Aufgabenrubrik in *Natur und Kultur* weiter und verfasste dort auch längere Beiträge. Einige dieser Beiträge zeugen von einer dem Nationalsozialismus deutlich zugeneigten Gesinnung. Dazu gehören beispielsweise der mit einer Philippika gegen Einstein beginnende Beitrag „Der Kampf um die Relativitätstheorie“ (*Natur und Kultur* 31 [1934], 315ff. und 357ff.) und der Beitrag „Vom germanischem Sternenwissen“ (*Natur und Kultur* 32 [1935], 147–158). In Letzterem referierte Hofmann bestätigend und unterstützend die Thesen des angesehenen Ideologen der völkischen Bewegung Otto Sigfrid Reuter (1876–1945) und bezog sich dabei auf dessen Werk *Germanische Himmelskunde*. *Untersuchungen zur Geschichte des Geistes* (Lehmann, München 1934).

Hofmann wurde 1939 im Rahmen der Durchsetzung des Führerprinzips an der Preussischen Akademie der Wissenschaften zu Berlin auf die Empfehlung von Ludwig Bieberbach (aktiver Nationalsozialist und Begründer der nationalsozialistisch geprägten Zeitschrift *Deutsche Mathematik*), der Hofmann wohl als politisch zuverlässig einstufte (dazu gehörte insbesondere die Mitgliedschaft in der NSDAP, siehe hierzu die Personalakte im Archiv der Humboldt-Universität, Berlin, Sign. UK H 391) zum Leiter der Leibniz-Forschungsstelle berufen. Eine Reihe von Hofmanns Publikationen zu Leibniz erschienen später in der *Deutschen Mathematik*. Trotz seiner nationalsozialistischen Verstrickungen war Hofmanns Einfluss nach 1945 auf die Entwicklung der Mathematikgeschichte in Deutschland erheblich: er organisierte und leitete bis zu seinem Tod die Kolloquien zur Mathematikgeschichte am Mathematischen Forschungsinstitut Oberwolfach, die eine bis heute andauernde Tagungstradition begründeten.

Der Einfluss Wieleitners auf die nationalistische Haltung Hofmanns lässt sich auf Basis des nachgelassenen Briefwechsels nicht eindeutig bestimmen. Klar ist jedoch, dass Wieleitners eigene Haltung zur Diskussion um die Leistungen von Leibniz im Kontext der Entwicklung der Differenzial- und Integalrechnung von einer Tendenz zur nationalistischen Instrumentalisierung geprägt war. Dies zeigte sich unter anderem in These 4 seiner bereits zitierten Probevorlesung: „Unserem Leibniz Unwahrhaftigkeit (oder auch nur ‚Vergesslichkeit‘) in der Berichterstattung vorzuwerfen, ist durchaus ungerechtfertigt“ (hier zitiert nach Hofmann 1933: 211). Dieser Satz inmitten sachlich formulierter wissenschaftlicher Inhalte nimmt sich etwas seltsam aus und bezieht sich – ohne, dass darauf verwiesen wird – offensichtlich auf die durch die Publikation von James M. Child 1920 (*The Early Mathematical Manuscripts of Leibniz*, Chicago und London, 1920) publizierte These, Leibniz habe die Ideen zu seiner Lehre von Isaac Barrow übernommen. In der *Geburt der Mathematik* äußerte sich Wieleitner zu Child zwar gemäßigt: „Wir würden auf diese Einzelheiten vielleicht auch kein so großes Gewicht legen, wenn nicht im Jahre 1920 ein Engländer, J. M. Child, […] zu beweisen versucht hätte, dass Leibniz wirklich die Grundlagen seiner Lehre Barrow entnommen habe. Die Frage selbst wollen wir nicht entscheiden, jedoch betonen, dass Child selbst sagt, auch wenn Leibniz nichts Anderes getan hätte als Barrow ins Analytische zu übersetzen, wäre sein Verdienst groß genug.“ (Wieleitner 1925b: 48). Jedoch bemüht sich Wieleitner auf den nachfolgenden Seiten intensiv um eine „Ehrenrettung“ von Leibniz. Ebenso beruht am Ende des Bandes seine Argumentation zur Bedeutung der Mathematik für philosophische Fragestellungen (1925b: 64 f.) im Grunde überwiegend auf Leibniz, ist von rhetorischem Charakter im Sinne einer Relevanzinszenierung und liefert keine fundierte Analyse zu diesem Themenkomplex.

## Mathematik und Medien

Die Tatsache, dass Wieleitner seit 1909 in *Natur und Kultur* dauerhaft eine Rubrik für Mathematische Aufgaben etablieren konnte, gründete unter anderem im Charakter dieser Zeitschrift, die sich an einer Synthese von erzählender Wissenschaftsvermittlung und wissenschaftlicher Berichterstattung versuchte (Daum 1998: 370–376).

In den vor etwa 1880 gegründeten wissenschaftsvermittelnden Medien wurde die Mathematik hingegen in der Regel marginalisiert, wenn nicht sogar aktiv ausgegrenzt. So distanzierte man sich 1868 in der Zeitschrift *Sirius* explizit von der „herablassenden Mandarinensprache mathematischer Formeln“, um zu einer „wahrhaft populären Darstellung“ zu gelangen.^19^ Zwar fanden publikumswirksame Einzelthemen wie die vierdimensionale Geometrie ihren Weg sogar in eine breitenwirksame illustrierte Zeitschrift wie die *Gartenlaube *(Volkert 2018: 133–181), jedoch erst die in den 1880er Jahren entstehenden, „akademisch Gebildete“ (Daum 1998: 366) adressierenden und damit der Ausdifferenzierung wissenschaftlicher Disziplinen Rechnung tragenden Zeitschriften wie die *Naturwissenschaftliche Wochenschrift* und die *Naturwissenschaftliche Rundschau* boten der Mathematik die Möglichkeit, sich in überschaubarem Umfang kontinuierlich zu präsentieren.

In der 1887 gegründeten, vor allem von Lehrern rezipierten *Naturwissenschaftlichen Wochenschrift *verwies im dritten Band des Jahrganges 1888 eine Notiz explizit auf das Vorhaben, „in längeren oder kürzeren Zwischenräumen auch mathematische Mitteilungen, Besprechungen mathematischer Werke, sowie grössere oder kleinere Berichte über neue Untersuchungen aus diesem Gebiete zu bringen“, um „damit die grösste mögliche Vielseitigkeit erreicht zu haben und den Wünschen vieler Leser zu entsprechen“^20^.

Inhaltlich verantwortlich zeichnete dafür der damals noch an seiner mathematischen Dissertation arbeitende August Gutzmer (1860–1924), der im Zeitraum von 1904–1907 Vorsitzender der Kommission für den mathematischen und naturwissenschaftlichen Unterricht war und 1905 zum ordentlichen Professor der Mathematik an die Universität Halle-Wittenberg berufen wurde. Mit Unterstützung der Pädagogen und Mathematiker Hermann Schubert (1848–1911) und Victor Schlegel (1843–1905) setzte er sich für eine regelmäßige Präsenz in erster Linie den mathematischen Unterricht betreffender Themen ein. Mit dem Verlagswechsel der Zeitschrift und der damit einhergehenden Neuausrichtung auf ein breiteres Publikum nach 1900 (Schirrmacher 2008b: 76) nahmen die mathematischen Beiträge zahlenmäßig etwas ab und beschränkten sich in den letzten Jahren vor der Einstellung der Zeitschrift 1922 auf Buchbesprechungen.

Bei der zwischen 1886 und 1912 beim Verlag Friedrich Vieweg & Sohn erscheinenden *Naturwissenschaftlichen Rundschau* sorgte Emil Lampe (1840–1918) seit etwa 1890 als Mitarbeiter und ab 1901 bis 1912 als Mitherausgeber für eine facettenreiche Darstellung der Mathematik. Neben der Einwerbung von Originalbeiträgen und Rezensionen trug Lampe selbst durch eine Reihe historischer Darstellungen im Kontext von Nachrufen^21^ auf Mathematiker*innen zur Vermittlung mathematischer Inhalte bei. Lampe, der seit den 1870er Jahren sowohl als Lehrer an der Preußischen Kriegsakademie in Berlin als auch Professor an der Technischen Hochschule Charlottenburg tätig war, erbrachte eine Vielzahl kommunikativer Serviceleistungen für die Disziplin. Er war nicht nur langjähriger Redakteur des Referateorgans *Jahrbuch für die Fortschritte der Mathematik*, sondern verfasste auch zahlreiche Rezensionen mathematischer Lehrbücher für die *Deutsche Literaturzeitung, Fortschritte der Physik* und das *Archiv der Mathematik und Physik*.

Eine langfristig erfolgreiche Neugründung des ausgehenden 19. Jahrhunderts war *Die Umschau *(gegründet 1897), die mit einer vergleichsweise hohen Auflage von etwa 15.000–20.000 Exemplaren in der Weimarer Zeit ein breites Themenspektrum unter Einbeziehung der Technik abdeckte (Picht 2010; Schirrmacher 2008a). Neben Siegmund Günther und dem Schriftsteller und Lehrer für Mathematik und Naturwissenschaften Kurd Laßwitz (1848–1910) lieferte vor allem Ernst Wölffing (1864–1933), zunächst Lehrer und später auch außerplanmäßiger Professor an der Technischen Hochschule Stuttgart, eine Reihe von Hauptartikeln und Buchbesprechungen. Dabei äußerte er sich entlang von Spezialthemen sowohl zur Relevanz einer theoretischen mathematischen Ausbildung der Techniker*innen als auch zur allgemeinen gesellschaftlichen Bedeutung der Mathematik. Nach 1909 reduzierte Wölffing seine Mitarbeit bei der *Umschau*, was den Stellenwert von Wieleitners Tätigkeit bei *Natur und Kultur *umso mehr erhöhte. Zumal die nach der Jahrhundertwende entstehenden naturkundlichen Magazine mit sehr großer Auflage, wie etwa der *Kosmos* (gegründet 1904) oder die 1925 gegründete *Koralle* (Schirrmacher 2008a), aus konzeptionellen Gründen der Mathematik nur wenig Raum ließen.

Eine weitere Folge der nach der Jahrhundertwende fortschreitenden Ausdifferenzierung der Druckmedien war auch die bereits oben erwähnte, 1913 erstmals erscheinende Zeitschrift *Die Naturwissenschaften*. Als Arnold Berliner (1862–1942) im August 1912 dem Springer-Verlag die Gründung einer fächerübergreifenden Zeitschrift mit dem entsprechenden Titel vorschlug, ging es ihm um die Etablierung eines Mediums, das „jeden naturwissenschaftlich Thätigen“ darüber orientieren solle, was ihn „außerhalb seines eigenen Faches interessiert“. Dieses Konzept entsprach der Intention der Neugründungen des ausgehenden 19. Jahrhunderts, wurde aber nach der Jahrhundertwende nicht mehr konsequent umgesetzt. Die Mathematik schloss Berliner dabei als Gegenstandsbereich in seinem Schreiben an Ferdinand Springer explizit aus, weil sie „zu speziell“ sei.^22^ Dass sie bereits in den ersten Bänden von *Die Naturwissenschaften* doch zumindest in geringem Umfang und überwiegend über Buchbesprechungen berücksichtigt wurde, war wohl der Tatsache geschuldet, dass die Finanzierung der Zeitschrift durch die gegen ein Entgelt erfolgte Übernahme der Abonnent*innen der eingestellten *Naturwissenschaftlichen Rundschau *ermöglicht wurde (Sarkowski 1992: 192ff.). Zum Sprachrohr des weltweit anerkannten mathematischen Zentrums in Göttingen entwickelten sich *Die Naturwissenschaften* erst nach 1918 durch die engagierte Zusammenarbeit von Berliner und Richard Courant (1888–1972), dem Gründer und ersten geschäftsführenden Direktor des Mathematischen Institutes der Universität Göttingen (gegründet 1922).

Einen wesentlichen Beitrag zur Mathematikvermittlung an nichtakademische Öffentlichkeiten leistete seit etwa 1910 neben Wieleitner in erster Linie Walther Lietzmann, der nach seiner Promotion 1904 in Mathematik über vierzig Jahre zunächst in Barmen, dann in Jena und schließlich in Göttingen im Schuldienst tätig war. In enger Zusammenarbeit mit Felix Klein wirkte er maßgeblich an der Reformbewegung des Mathematikunterrichts für höhere Schulen mit und galt mit seinem 1916 erschienenen Hauptwerk zur Methodik des mathematischen Unterrichts als Begründer der Mathematikdidaktik. Zunächst seit 1920 als Privatdozent und später als Honorarprofessor vertrat er an der Universität Göttingen die Pädagogik der exakten Naturwissenschaften. Darüber hinaus gelang es ihm mit der bereits genannten Buchreihe *Mathematisch-Physikalische-Bibliothek* seit 1911 und bis nach 1945 ein dauerhaft tragfähiges Format zu etablieren mit der Zielsetzung, „allen denen, die Interesse an der Mathematik im weitesten Sinne des Wortes haben, es in angenehmer Form zu ermöglichen, sich über das gemeinhin in den Schulen Gebotene hinaus zu belehren und unterrichten“ (Reihenbeschreibung beispielsweise in Lietzmann 1912). Der Großteil der Neuerscheinungen der insgesamt etwa 100 Bände umfassenden Reihe datierte auf die Jahre vor 1932, während nach 1939 nur noch Wiederauflagen zu verzeichnen sind. Das handliche Format, die Beschränkung des Umfangs auf etwa 80 Druckseiten und der damit einhergehende günstige Preis (1924 betrug dieser 1,20 RM, was etwa 3,60 Euro entspricht) bescherten dem Unternehmen mit etwa einer halben Million verkaufter Exemplare einen anhaltenden Erfolg (Lietzmann 1960: 91 f.).

Die Werke der Sammlung wurden von Verlag und Herausgeber in vier Gruppen eingeordnet: (1.) Biografisch-Historisches, (2.) Arithmetik, Algebra und Analysis, (3.) Geometrie, (4.) Angewandte Mathematik. Mehr als ein Drittel der Bände widmeten sich dabei den Anwendungen der Mathematik in der Technik und der Wirtschafts- und Finanzmathematik, während der Bereich Mathematik und Kultur (siehe etwa Geschichte der Mathematik oder Mathematik und Malerei) lediglich von etwa 15 Prozent der Publikationen abgedeckt wurde. Diese Schwerpunktsetzung spiegelte Lietzmanns Vorstellung von den Aufgaben „gemeinverständlicher mathematischer Literatur“ als Mittel „staatsbürgerlicher Erziehung“. Dazu gehörten aus seiner Sicht neben der Förderung des mathematischen Denkens in der Allgemeinheit vor allem eine Steigerung des Bewusstseins für die Relevanz des Beitrags der Mathematik zur Entwicklung von Wirtschaft und Technik (Lietzmann 1921: 52 f.).

Diese Auffassung setzte er auch in der *Zeitschrift für den mathematischen und naturwissenschaftlichen Unterricht (ZMNU)* um, bei der er seit 1914 als Mitherausgeber tätig war. Damit einher ging eine deutliche Profiländerung dieses Mediums, das sich ursprünglich der Integration von Pädagogen unterschiedlicher Schulformen verschrieben hatte (Teubner et al. 1914). Unter dem Einfluss Lietzmanns wurden die Themen der Reformbewegung in erster Linie des mathematischen Unterrichts, wie die Etablierung der Infinitesimalrechnung und die Stärkung der angewandten Mathematik, in den Vordergrund gerückt. Darüber hinaus sorgte Lietzmann insbesondere seit Beginn der 1920er Jahre auch für die umfassende Einbindung von Beiträgen, die seinen Kriterien einer allgemein verständlichen Darstellung genügten. Damit gelang ihm eine deutliche Erweiterung der Leserschaft, die – wie den mit „Sprechsaal“ überschriebenen Rubriken der Zeitschrift und der Korrespondenz aus seinem Nachlass^23^ zu entnehmen ist – unter anderem Jurist*innen, Ingenieur*innen und Hobby-Mathematiker*innen aus allen Bereichen umfasste.

Lietzmanns und Wieleitners komplementäre Schwerpunkte im Kontext medial vermittelter Relevanzinszenierungen der Mathematik ergänzten sich nicht nur im Rahmen der *Mathematisch-Physikalischen Bibliothek* oder der *ZMNU*, wo auch Wieleitner regelmäßig publizierte. Sie fanden vor allem eine gemeinsame institutionelle Basis in dem 1921 gegründeten *Reichsverband deutscher mathematischer Gesellschaften* (kurz*: Mathematischer Reichsverband*), der sich in seiner Satzung die Aufgabe stellte, „das Verständnis für die praktische und kulturelle Bedeutung der Mathematik zu verbreiten und sie im öffentlichen Leben zu vertreten. Er will ferner eine enge Verbindung zwischen Forschung, Unterricht und Praxis anstreben, zur gegenseitigen Förderung und Anregung.“^24^ Neben einer Reihe regionaler mathematischer Vereine gehörten zu den Gründungsmitgliedern als nationale Verbände die *Deutsche Mathematiker-Vereinigung*, der *Verein zur Förderung des mathematischen und naturwissenschaftlichen Unterrichts* und der *Deutsche Verein für Versicherungswirtschaft.* Für die Geschäftsführung verantwortlich zeichnete ein in Berlin ansässiger Arbeitsausschuss, dem ein aus Vertreter*innen der einzelnen Vereine bestehender Beirat zugeordnet war (Tobies 1993: 248 f.). Heinrich Wieleitners Wahl in den Beirat dokumentiert der Sitzungsbericht des Arbeitsausschusses vom 20.10.1924,^25^ der auch Lietzmann als Mitglied ausweist. Weitere Sitzungsberichte belegen, dass der Arbeitsausschuss es als wichtige Aufgabe ansah, die Darstellung der Mathematik in Zeitungen, Zeitschriften und anderen Publikationen zu beobachten. Ziel war offensichtlich, sowohl für eine verbesserte öffentliche Sichtbarkeit der Disziplin zu sorgen als auch gegebenenfalls durch geeignete Stellungnahmen zügig auf negative Berichterstattung zu reagieren.^26^

Eine Vielzahl der sich um und nach 1900 neu etablierenden wissenschaftsvermittelnden Zeitschriften und Buchreihen distanzierte sich sowohl in ihrer Selbstbeschreibung als auch intentional von dem das 19. Jahrhundert dominierenden Begriff der Popularisierung. Schirrmacher (Schirrmacher 2008b) leitet unter anderem daraus zu Recht eine Kritik an dessen Verwendung für die Beschreibung von Kommunikationsprozessen zwischen Wissenschaft und Öffentlichkeit im 20. Jahrhundert ab, die sich in ihren vielfältigen Erscheinungsformen keineswegs mehr auf die Vermittlung wissenschaftlicher Ergebnisse beschränkte. Vielmehr nahm sie sich auch der Darstellung von „Produktionsprozessen der Wissenschaft und deren Normen“ an (Schirrmacher 2008b: 81).

Diese Tendenz spiegelte neben der inhaltlichen auch eine materiale Ausdifferenzierung von Druckmedien. So ist neben der Neuausrichtung des Zeitschriftenwesens nach der Jahrhundertwende auch eine Zunahme von wissenschaftsvermittelnden Buchreihen zu verzeichnen (dazu gehörte auch *Wissen und Wirken*, der Publikationsort von Wieleitners hier verhandelter Monografie), die geeignet waren, disziplinübergreifende wissenschaftstheoretische Fragen jenseits der Fachwissenschaft zu verhandeln.

Gerade diese strukturellen Veränderungen der Medienlandschaft schufen jedoch erst die Bedingungen, die der Mathematik eine Präsentation als autonome Disziplin ermöglichten. In diesem Sinne war die Popularisierung der Mathematik als Phänomen des 19. Jahrhunderts tatsächlich ein sowohl für die Disziplin als auch im gesellschaftlichen Diskurs marginales Phänomen.

## Heinrich Wieleitner und die Mathematikkommunikation in der Weimarer Zeit

Die Bedeutung der Mathematikgeschichte für die Ausbildung angehender Mathematiker*innen und Mathematiklehrer*innen seit der Wende zum 20. Jahrhundert zeigte sich nicht nur in den entsprechenden von Wieleitner mitgestalteten Bänden der *Sammlung Schubert*. So setzten sich etwa auf der institutionellen Ebene Mathematiker*innen, Mathematikhistoriker*innen und -pädagog*innen im Rahmen des Internationalen Mathematiker-Kongresses 1904 in Heidelberg dafür ein, den universitären mathematischen Unterricht durch mathematikhistorische Lehrveranstaltungen zu vervollständigen. Zu den aus Deutschland stammenden Unterstützer*innen dieser Bewegung gehörten unter anderem Ernst Wölffing und Emil Lampe (Fauvel & van Maanen 2002: 91 f.). Beide erkannten und nutzten im Rahmen ihrer Tätigkeit als Mitarbeiter der oben genannten wissenschaftsvermittelnden Zeitschriften auch das Potenzial des historischen Erzählens zur Kommunikation jenseits der Fachwissenschaft.

Welche Aspekte dieses Potenzial im disziplinären Vergleich insbesondere für die Mathematik konstituierten, formulierte Felix Klein prägnant in der Einleitung zu den* Vorlesungen zur Entwicklung der Mathematik im 19. Jahrhundert* (Klein 1926: 1 ff.; der Text erschien posthum und erschien in einer von Richard Courant und Otto Neugebauer herausgegeben Sammlung von Kleins in engstem Kreis gehaltenen Vorlesungen während des Ersten Weltkriegs). Die „schroffe Abgeschlossenheit“ der Mathematik lasse bei deren Präsentation für die Allgemeinheit lediglich zu, „ein Bild davon zu geben, was die Mathematiker etwa treiben“ (Klein 1926: 1). Die historische Entwicklung in den Vordergrund zu stellen, ermögliche es, das Interesse der Leser*innen zu wecken, ohne sie übermäßig mit schwer verständlichen systematischen Einzelheiten zu belasten. Darüber hinaus werde „die Betonung der Einwirkung der Mathematik auf ihre Nachbargebiete und eine lebendige Darstellung ihrer Beziehungen zu unserem gesamten Kulturleben für jeden gebildeten Leser irgendeinen Anknüpfungspunkt bilden“ (Klein 1926: 2). Gleichzeitig stellte er fest, dass keine „populäre Darstellung“ der Mathematik ohne eine „pia fraus“ (lat. frommer Betrug) auskomme, da dem/der Rezipienten/*in nur suggeriert werde, er/*sie sei dem Gegenstand tatsächlich nahegekommen, wo er doch nur seine äußere Form erfasst habe. Der Einsatz mathematikhistorischer Formate erschien dabei nicht nur Klein als besonders guter Kompromiss zwischen „Vermittlung und Verrat“ (Heumann 2014: 22) – ein Gegensatzpaar, das die ambivalente Haltung nicht nur einer Reihe von Mathematiker*innen gegenüber breitenwirksamer Wissensvermittlung treffend beschreibt.

Heinrich Wieleitner gelang es in seinen Publikationen zur Geschichte der analytischen Geometrie und der Infinitesimalrechnung seit Beginn der 1920er Jahre diesen Kompromiss in verschiedener Hinsicht erfolgreich zu realisieren. Zum einen dokumentierte er sehr klar, an welchen Stellen er der mathematischen Vorbildung seiner jeweiligen Leserschaft geschuldete Reduktionen des wissenschaftlichen Gegenstandes vornahm. Gleichzeitig boten seine Darstellungen in hinreichender Dichte Inhalte an, die mit geringen Vorkenntnissen gut nachvollziehbar waren, wobei seine Texte nicht nur passiv konsumierbares Wissen bereitstellten, sondern die Rezipient*innen zu aktiver Mitarbeit anregten.

Die historische Entwicklung der Analytischen Geometrie und der Infinitesimalrechnung erwies sich für diese Art der Integration mathematischer Formelsprache als besonders geeignet, denn sie ermöglichte etwa im Zusammenhang mit der griechischen Mathematik nicht nur eine Reihe leicht aktiv nachvollziehbarer Argumentationsketten. Gleichzeitig eröffnete sich auch ein Kontext, in dem schwer zugängliche, epistemisch zentrale Begriffe der Mathematik, wie etwa „Strenge“, „Beweis“ und „unendlich“, Anschlussfähigkeit erlangen konnten.

Die Entscheidung Wieleitners, diese Thematik für eine je unterschiedliche Leserschaft aufzubereiten, ist auch als Reaktion auf die Herausforderung zu werten, sowohl Kontinuität als auch Dynamik der Entwicklung mathematischen Wissens und mathematischer Praxis herauszuarbeiten und gleichzeitig den grundlegenden Konzepten der modernen Mathematik des 19. Jahrhunderts mehr positive Aufmerksamkeit zu sichern.

Die Fruchtbarkeit dieser Bemühungen war einerseits einer veränderten Medienlandschaft geschuldet, die sich von der Popularisierungsbewegung des 19. Jahrhunderts distanzierte und als Folge davon Formate bereitstellte, die der Darstellung mathematischer Wissensproduktion hinreichend Raum ließen. Gleichzeitig beförderte der sich seit der Wende zum 19. Jahrhundert entwickelnde und in den 1920er Jahren erstarkende sogenannte „Dritte Humanismus“ (Striewe 2011) den rezeptiven Erfolg von historischen Darstellung, die den Rückgriff auf antike Vorbilder in den Vordergrund stellten.

Wieleitner konnte so eine Reihe unterschiedlicher Rezipient*innengruppen ansprechen. Während die zweibändige *Geburt der modernen Mathematik *(1924b, 1925b) ein akademisch gebildetes Publikum adressierte, eigneten sich die für das *Mathematische Lesebuch* (1920, 1921a) verfassten Aufsätze dazu, die gleiche Thematik einem breiteren Leser*innenkreis wie etwa den Besucher*innen von Volkshochschulen und Fachschulen nahe zu bringen. Der in der Zeitschrift *Das Weltall* veröffentlichte Text „Die Geburt der modernen Mathematik“ (Wieleitner 1924a) vermittelte in komprimierter Form die Zielsetzungen der zweibändigen Buchausgabe, passte sie aber an die Erwartungen der Leserschaft der Zeitschrift an, indem er sich vor allem der Relevanz der analytischen Geometrie und der Infinitesimalrechnug für die Astronomie widmete. Die Adaptionen an die Bedürfnisse unterschiedlicher Leser*innenkreise führte zwar zu Bedeutungsverschiebungen in der Einordnung der identitätsbildenden Kernkompetenz der Mathematik, nämlich dem streng durchgeführten Beweis. Sie blieben jedoch immer in einem Rahmen, in dem gleichermaßen die Anwendungsfähigkeit der Mathematik und deren kulturelle Einbettung wie die Bedeutung der autonomen Entwicklung der Mathematik, jenseits philosophischer Überlegungen und gesellschaftlicher Nützlichkeitserwägungen, herausgearbeitet werden konnte. Dabei galt die Hervorhebung dieser Autonomie spätestens seit dem ausgehenden 19. Jahrhundert als Reaktion auf vielfältige Formen der Aneignung mathematischen Wissens durch Naturwissenschaften und Technik als wesentlicher Bestandteil des disziplinären Selbstverständnisses.

Die 1920er Jahre forderten dieses Selbstverständnis auf verschiedene Weise weiter heraus. Neben Spenglers *Untergang des Abendlandes *führte vor allem die öffentliche Diskussion um die Relativitätstheorie, deren Unzugänglichkeit häufig den zugrunde liegenden mathematischen Konzepten zugeschrieben wurde, zu einer kontroversen Wahrnehmung der Disziplin in akademischen und nichtakademischen Öffentlichkeiten (Hentschel 1990; Wazeck 2009; Flatau 2015). Gleichzeitig ging es im Zuge der Institutionalisierung der angewandten Mathematik um eine die Disziplin integrierende Bestimmung des Verhältnisses von reiner und angewandter Mathematik (Frühstückl 2018).

Die Mathematik begegnete diesen Herausforderungen durch kommunikative Aktivitäten auf unterschiedlichen Ebenen. Neben der geschilderten Institutionalisierung der Kommunikation durch den *Mathematischen Reichsverband* und der Anbindung nichtuniversitärer Vermittler*innen wie Wieleitner und Lietzmann an die Organisationen der Disziplin zeichnete sich die Weimarer Zeit dadurch aus, dass im Gegensatz zu den Jahren um 1900 zunehmend auch akademisch eingebundene Mathematiker*innen durch entsprechende Publikationen zur mathematischen Öffentlichkeitsarbeit beitrugen und dabei häufig entlang historischer Argumentationen die Stellung der Mathematik in der Kultur in den Blick nahmen.^27^

Insgesamt erlebte Mathematikkommunikation in den 1920er Jahren eine Intensivierung, die die These vom wissenschaftsfeindlichen kulturellen Klima der Weimarer Zeit insofern relativiert (hierzu auch Schirrmacher 2008a), als dass die ausdifferenzierte Medienlandschaft einer positiven disziplinären Selbstdarstellung der Mathematik durchaus Raum bot. Der Mathematikgeschichtsschreibung kam dabei eine wesentliche Rolle zu.

## Danksagung

Für die freundliche Bereitstellung des Nachlasses von Heinrich Wieleitner danke ich dem Archiv des Deutschen Museums in München, insbesondere Herrn Archivar Dr. Matthias Röschner. Danke auch an die stets hilfsbereiten Mitarbeiter*innen der Bildstelle des Deutschen Museums für die freundliche Betreuung vor Ort.

Für zahlreiche wertvolle Hinweise und Diskussionen danke ich Volker Remmert, Dawid Rowe, Erhard Scholz und Klaus Volkert, den Mitglieder*innen der Oberseminare für Geschichte der Mathematik in Mainz und Wuppertal, ebenso wie den anonymen Gutachter*innen, den Herausgeber*innen und der Redaktion von NMT.

Für die Unterstützung dieses Projekts durch Sachbeihilfen geht der Dank an die Deutsche Forschungsgemeinschaft.

## Footnotes

1 Systematisch untersucht wurden unter anderem folgende Zeitschriften und Buchreihen: *Sirius* (1868–1905), *Naturwissenschaftliche Rundschau* (1886–1912), *Naturwissenschaftliche Wochenschrift* (1887–1922), *Die Naturwissenschaften* (1913–1944), *Die Umschau* (1897–1943), *Unsere Welt* (1920–1945), *Das Weltall* (1900–1933), *Mathematisch-Physikalische Bibliothek, Weber’s illustrierte Katechismen, Aus Natur und Geisteswelt, Verständliche Wissenschaft, Sammlung Vieweg, Die Wissenschaft*. Ergänzend erfolgten punktuelle Sichtungen der Zeitschriften *Kosmos *(1904ff.), *Natur* (1910ff.) und *Deutsche Revue *(1880–1910) und der Tageszeitungen *Vossische Zeitung* und *Allgemeine Zeitung*.

2 Eine ausführliche Analyse dieser Ergebnisse wird derzeit in der in Vorbereitung befindlichen Monografie „Inszenierungen einer unsichtbaren Disziplin. Mathematikkommunikation im 19. und 20. Jahrhundert“ (erscheint voraussichtlich 2023 bei Springer) erarbeitet.

3 Für die biografischen Fakten zu Wieleitner siehe, sofern nichts anderes vermerkt ist, Bortolotti (1932); Dauben & Scriba (2002: 564–566); Hofmann (1932, 1933, 1934); Lietzmann (1932); Lorey (1932); Ruska (1932); Tropfke (1932); Vogel (1932a, b); Dictionary of Scientific Biographie 14: 336–337. Ein ausführliches Schriftenverzeichnis findet sich in Hofmann (1933).

4 Der umfangreiche Nachlass wurde dem Archiv des Deutschen Museums (DMA) dankenswerterweise von Menso Folkerts zur Verfügung gestellt und ist dort unter der Signatur NL 252 einzusehen.

5 Von Dyck arbeitete beispielsweise seit 1906 intensiv an einer Werkausgabe zu Johannes Kepler, die erst 1937 nach seinem Tod vollendet wurde; Lindemann publizierte neben mathematischen Fachbeiträgen auch zur Mathematikgeschichte, wie etwa ein Werk zur Geschichte von Polyedern und Zahlzeichen 1897.

6 DMA NL 252/034: Brief von B. Datta an H. Wieleitner vom 28. Mai 1929.

7 DMA NL 252/032: Brief von Ludwig Borchardt an Wieleitner vom 10. Mai 1927.

8 Siehe die verlagsseitige Reihenbeschreibung in Wieleitner 1924.

9 (1924b: 4).

10 Jurdant diskutiert hier, welchem literarischen Genre allgemeinverständliche wissenschaftsvermittelnde Literatur in Form nichtfiktionaler Texte über Wissenschaft zugeordnet werden könne und wählt die Autobiografie – nicht zuletzt wegen des Wahrheitsgehalts (im Vergleich zu fiktionalen Texten) und der Reflexivität dieser Textsorte.

11 Etwa Geburt, Wiedererweckung, Wesen u. ä.; bei mathematisch-technischen Passagen beispielsweise Geradstreckung für Rektifikation und Aufmessung für Quadratur.

12 Schreiben des Verlegers G. Braun an Wieleitner vom 10. August 1929, DMA NL2 52/032.

13 Zur Korrespondenz zwischen Wieleitner und Toeplitz siehe DMA, NL 252/052, hier der Brief vom 7. Juni 1925 von Toeplitz an Wieleitner.

14 Brief von Toeplitz an Wieleitner vom 16. April 1928, DMA, NL 252/052.

15 Brief von Toeplitz an Helmut Hasse vom 27. Oktober 1928, siehe die Transkriptionen des Briefwechsels Hasse-Toeplitz von Peter Roquette: https://www.mathi.uni-heidelberg.de/~roquette/Transkriptionen/manutrans.html.

16 Siehe oben genannter Brief von Toeplitz an Hasse.

17 Brief von Toeplitz an Wieleitner vom 16. April 1928, DMA, NL 252/052: „Ich würde das wertvollste Resultat derselben [der Habilitation] darin erblicken, wenn sie Ihnen durch vermehrte Berührung mit den mathematischen Studenten Schüler in historicis einbrächte.“

18 Brief von Ludwig Bieberbach (1886–1982), dem damaligen Schriftführer der Deutschen Mathematiker-Vereinigung, vom 13.09.1930, DMA, NL 252/032.

19 Sirius 1868 (1): 1.

20 *Naturwissenschaftliche Wochenschrift*, 1888 (3): 79.

21 Siehe hierzu beispielsweise die Nachrufe von Emil Lampe in der *Naturwissenschaftlichen Rundschau* zu Leopold Kronecker (1892), Eduard Kummer (1893), Karl Weierstraß (1897), Sophus Lie (1899), Charles Hermite (1901), Luigi Cremona (1903) und den Beitrag zu Gustav Lejeune Dirichlets 100. Geburtstag 1906.

22 Brief von Arnold Berliner an Ferdinand Springer vom 06.08.1912, Archiv des Springer-Verlags, Korrespondenz: Die Naturwissenschaften – Arnold Berliner.

23 Nachlass Lietzmann, Stadtarchiv Göttingen, Dep89, D2.

24 Satzung des Mathematischen Reichsverbandes siehe Jahresbericht der Deutschen Mathematiker-Vereinigung 1922 (31), Abteilung (2), 124 f.

25 Siehe den entsprechenden Sitzungsbericht im Nachlass Wieleitners, DMA, NL 252/049.

26 Siehe hierzu die Protokolle der Sitzungen des Mathematischen Reichsverbandes im Nachlass Wieleitner, DMA, NL 252/049 von 1924 bis 1930 und vereinzelte Protokolle im Nachlass Lietzmann, Stadtarchiv Göttingen, Dep 89, F2.

27 Z. B. Otto Toeplitz: *Von Zahlen und Figuren* (1932) oder Hilda Pollaczek-Geiringer: *Die Gedankenwelt der Mathematik *(1922), vgl. hierzu Bergmann & Epple (2009: 172–181) und den zuvor zitierten Aufsatz von Schirmeier (2008) zu *Die Naturwissenschaften*.
